# The Appropriateness of Empirical Uses of Antibiotics Based on Antimicrobial Susceptibility Results for Inpatients at a Tertiary Hospital in Saudi Arabia

**DOI:** 10.3390/antibiotics13090824

**Published:** 2024-08-30

**Authors:** Yasser Almogbel, Mugahid A. Mobark, Masaad S. Almutairi, Faisal S. Almogbel, Syed I. Rabbani, Sultan Alhathloul, Shada Alamro, Lateefah Alatallah

**Affiliations:** 1 Department of Pharmacy Practice, College of Pharmacy, Qassim University, Buraydah 51452, Saudi Arabia; mu.mohammed@qu.edu.sa (M.A.M.); mas.almutairi@qu.edu.sa (M.S.A.); 2 Family Medicine Academy, Qassim Health Cluster, Buraydah 52367, Saudi Arabia; falmogbel@gmail.com; 3 Preventive Medicine, King Fahd Specialist Hospital, Buraydah 52366, Saudi Arabia; 4 Department of Pharmacology and Toxicology, College of Pharmacy, Qassim University, Buraydah 51452, Saudi Arabia; s.rabbani@qu.edu.sa; 5 PharmD Graduates, Department of Pharmacy Practice, College of Pharmacy, Qassim University, Buraydah 51452, Saudi Arabia; sultanalhathloul@gmail.com (S.A.); shadaalamro@gmail.com (S.A.); lateefahpharmacy@gmail.com (L.A.)

**Keywords:** antibiotic, antibacterial agent, appropriateness, empirical uses of antibiotics

## Abstract

The optimal use of antibiotics represents a cornerstone in controlling antibiotic resistance. Strategies such as antibiotic stewardship programs (ASPs) have been developed to influence the rational use of antibiotics. This study evaluated the appropriateness of the empirical use of antibiotics based on antibiotic susceptibility results with the aim of participating effectively in improving local ASPs. In a cross-sectional study conducted at a tertiary hospital in Saudi Arabia, 500 inpatients received empirical antibiotics, and their culture and sensitivity results were included. The appropriateness of the empirical use of antibiotics was determined based on their alignment with the culture and sensitivity results. More than half of the participants (56.4%) were men, and nearly half (43%) were over 61 years old. The empirical uses of antibiotics were appropriately prescribed in 58% of the patients. Ciprofloxacin and ceftriaxone were the most prescribed antibiotics, while vancomycin, piperacillin–tazobactam, and tigecycline were the most appropriately prescribed antibiotics. *E. coli* was the main microorganism isolated in the susceptibility results and was appropriately prescribed in 59% of the patients. The highest microbial sensitivity was observed for linezolid, vancomycin, and tigecycline. Antibiotics were appropriately prescribed empirically in more than half of the participants. Activating interventional ASP is crucial to fill the gap in prescribing antimicrobials. Considering the expected type of organisms and the local susceptibility pattern is likely to yield a more appropriate empirical use of antibiotics.

## 1. Introduction

Infectious diseases were the leading cause of morbidity and mortality in human populations before the introduction of antibiotics [1]. Since their introduction, antibiotics have been widely prescribed, and currently they are the most frequently prescribed drugs in hospitalized patients worldwide [2]. However, this increasingly widespread use, especially irrational use, has led to the appearance of antibiotic resistance, representing one of the top emerging problems in modern medicine concerning infection control [3]. Infections caused by multidrug-resistant bacteria are associated with a higher incidence of mortality and prolonged hospital stays compared to those caused by susceptible bacterial infections [4,5]. Antibiotic resistance is so serious that the World Health Organization (WHO) published a global strategy for its containment and a resolution on promoting the rational use of medicines [6,7].

To encourage the optimal use of antibiotics, many strategies have been developed, often known as antibiotic stewardship programs (ASPs), which have been further categorized as educational and restrictive strategies. Both aim to reduce the use of antibiotics and thus the cost of medication, in addition to maintaining the quality of care [8,9].

In 2017, the World Health Organization (WHO) developed the Access, Watch, and Reserve (AWaRe) classification system of antibiotics as part of ASPs. The WHO AWaRe groups antibiotics according to their spectrum of activity and potential for developing resistance [10]. The Access group contains antibiotics used in the first- and second-line treatment of infections; they are active against a wide range of commonly encountered susceptible pathogens and have a lower resistance potential than antibiotics in the other groups. The Watch group contains broad-spectrum antibiotics with a higher potential for developing resistance. The Reserve group contains last-resort antibiotics used for multidrug-resistant infections [11]. 

In a meta-analysis study, the prevalence of multidrug-resistant organisms and mortality rates were significantly associated with the inappropriate empirical use of antibiotics [12]. To achieve the best approaches for antibiotic stewardship programs, the Infectious Diseases Society of America (IDSA) and the Society for Healthcare Epidemiology of America (SHEA) have provided recommendations that are categorized into intervention, optimization, microbiology, and laboratory diagnostics issues; measures for reflecting on, assessing, and improving ASPs and their interventions; and, finally, the concept of special populations [13].

In Saudi Arabia, a pharmaceutical strategic plan was started in 2013 [14]. In 2014, the central committee at the Ministry of Health published an ASP manual that incorporates the empirical use of therapy for common infectious diseases in Saudi Arabia; data collection on certain selected antibiotics, antimicrobial sensitivities patterns, restricted and controlled antibiotics; and policies and procedures for prescribed antibiotics [15]. The fundamental applications of national ASPs started in 2015 in more than ten regions and more than 40 hospitals [16].

This study investigated the appropriateness of the empirical use of antibiotic prescription based on the antimicrobial susceptibility results from inpatients at a tertiary hospital in Saudi Arabia, King Fahad Specialist Hospital (KFSH), as well as providing research-based data about local antimicrobial susceptibility patterns with the aim of participating in improving and updating future local ASPs.

## 2. Results

The demographic characteristics of the 500 patients are presented in Table 1. The percentage of men was found to be 56.4% (282). In the age distribution, almost half of the patients were aged above 61 years (43%; 215), followed by those age 41–60 years (31.6%; 158) and 18–40 years (23.4%; 117), and the smallest group was <18 years (2%; 10).

Table 2 illustrates the frequency of the empirical use of antibiotics and their appropriateness. Among the 500 antibiotics used empirically, 58% were appropriately prescribed. The most frequently prescribed antibiotics were ciprofloxacin (56), followed by ceftriaxone (52), amoxicillin, clavulanate, and ceftazidime (49 each); and the least prescribed was cefuroxime (6). The highest percentage of appropriateness was seen with vancomycin (94%), followed by piperacillin–tazobactam and tigecycline (73% each), and then ceftriaxone (71%). The least appropriateness was seen with imipenem (14%). 

The most common type of infection in this study was urinary tract infection (UTI) (123, 46.6%), followed by sepsis (100, 20%). The analysis to determine the appropriateness of the empirical use of antibiotics showed that abdominal infection had the highest percentage of appropriate treatment with empirical therapies, 6/8 (75%); followed by lower respiratory tract infection (LRTI), 50/69 (72%); sepsis, 68/100 (68%); and upper respiratory tract infection (URTI), 15/23 (65%). However, the most inappropriate empiric antibiotic prescriptions were seen in burns, 3/5 (60%); and central nervous system (CNS) infections, 10/18 (56%) (Table 3).

Table 4 summarizes the appropriateness of the empirical use of antibiotics according to the type of organism. The data suggested that *Escherichia coli* (*E. coli*) (167, 33.4%) was the microorganism causing the most infections, followed by *Staphylococcus aureus (S. aureus)* (74, 14.8%), then *Acinetobacter baumannii* (66, 13.2%), *Enterococcus avium* (42, 8.4%), and *Enterobacter cloacae* (28, 5.6%). The highest appropriateness of the empirical use of antibiotics was observed in *Enterobacter aerogenes,* 6/8 (75%); followed by *A. baumannii,* 46/66 (70%), *A. lowffii,* 9/14 (64%), *S. aureus,* 46/74 (62%); and *Citrobacter koseri,* 12/20 (60%). Methicillin-resistant *S. aureus* (MRSA) and *E. coli* were appropriately treated in 16/27 (59%) and 90/167 (54%) of the cases, respectively. 

Table 5 shows the appropriateness of the empirical use of antibiotics according to comorbidities. The three most common comorbidities recorded in patients were diabetes mellitus ( (35.6%; 93), chronic renal disease (23.4%; 61), and hypertension (19.9%; 52). The most appropriate empirical use of antibiotics was observed with chronic renal failure 54/61 (88.5%), asthma 14/16 (87.5%), diabetes 81/93 (87%), epilepsy 6/7 (85.7%), hypothyroidism 9/11 (82%), congestive heart failure 5/7 (71.4%), and ischemic heart disease 10/14 (71.4%). The least appropriateness was recorded for hypertension 32/52 (61.5%). 

Table 6 indicates the antimicrobial susceptibility patterns to different pathogens. The three most frequently used antibiotics in the sensitivity test were ciprofloxacin, meropenem, and cefepime. The highest percentage of microbial sensitivity was observed for linezolid 86/88 (98%), vancomycin 96/100 (96%), and tigecycline 133/144 (92%), while ceftazidime 148/362 (41%) had the lowest sensitivity. In contrast, cefepime, oxacillin, levofloxacin, ciprofloxacin, and ceftazidime were found to be resistant in more than 50% of the sensitivity testing.

**Figure 1:**
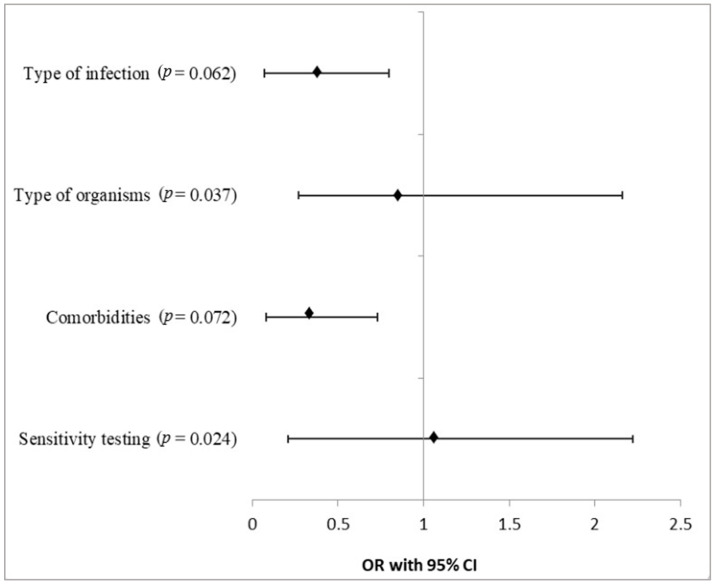
Forest plot of logistic regression analysis of the appropriateness of the empirical use of antibiotics against various variables. Statistics: The chi-square test was used for logistic regression analysis. The *p*-value indicates a comparison among groups.

Figure 1 demonstrates the logistic regression analysis in the form of a forest plot. The odds ratios for different variables, such as type of infection, type of organism, comorbidities, and sensitivity testing, were found to be 0.35, 0.91, 0.29, and 1.03, respectively. The analysis of the data suggested a greater appropriateness of the empirical use of antibiotics for variables such as sensitivity testing (OR = 1.03, 95% CI = 0.82–1.19) and type of organism (OR = 0.91, 95% CI = 0.64–1.25), and the *p*-value for these were less than 0.05. However, the association of the appropriateness of the empirical use of antibiotic therapy for type of infection (OR = 0.35, 95% CI = 0.28–0.45) and comorbidities (OR = 0.29, 95% CI = 0.21–0.44) was found to be mild-to-moderately correlated. The *p*-value for these variables was more than 0.05.

## 3. Discussion

Nowadays, antibiotic resistance is a global public health catastrophe that has become a health challenge for the WHO and antimicrobial stewardship programs [17]. The WHO developed the AWaRe classification of antibiotics in order to control and reduce the spread of antimicrobial resistance and decrease the impact of this resistance on public health. Although access groups for antibiotics are widely available and relatively safe, their irrational use increases the possibility of more antimicrobial resistance. Watch group antibiotics have higher resistance compared to those of the Access group, and the Reserve group is the last weapon against bacteria that should be used only upon the failure of the others [18,19]. 

This cross-sectional, hospital-based study investigated the appropriateness of empirical use of antibiotics according to the antimicrobial susceptibility results from 500 inpatients at KFSH in the Qassim region. The empirical use of antibiotics was slightly higher for men (56.4%). In the age distribution, the majority of patients were over 61 years old (43%), reflecting a greater susceptibility of elderly people to bacterial infection. A study targeting Gram-negative bacterial infection in the Aljouf region of Saudi Arabia showed that more than half of bloodstream infections occurred in patients aged ≥ 60 years [20]. As reported previously, aging-related factors like dementia and frailty play a role in susceptibility to infections in elderly people [21].

In this study, 58% of the antibiotics used empirically were appropriately prescribed. This percentage of appropriate use is expected to increase with the use of two antibiotics instead of only one, as reported previously [22]. Similarly, in a study conducted in China, the empirical use of antibiotics was inappropriate in 42.6% of the patients based on culture and sensitivity results, and the empirical use of antibiotics was appropriate but unnecessarily broad spectrum in 29.3% of the patients [23]. 

In addition, this study showed that the antibiotic the was most prescribed empirically was ciprofloxacin, followed by ceftriaxone, amoxicillin and clavulanate, and ceftazidime, and the least prescribed was cefuroxime. Although it was highly prescribed, ciprofloxacin was appropriately used in only 50% of patients. The frequent use of ciprofloxacin can be explained by the higher frequency of UTI in the studied sample. A previous study conducted in King Faisal Specialist Hospital (KFSH) reported that trimethoprim and sulfamethoxazole (TMP+SMX) and ciprofloxacin were the most frequent empirically prescribed antibiotics for UTI [24]. Furthermore, this study found that the highest percentage (94%) of appropriate empirical use of antibiotics was seen with vancomycin. This finding is attributed to the higher bacterial susceptibility to vancomycin, even in MRSA. As reported in the western region of Saudi Arabia, almost all isolated MRSAs were susceptible to vancomycin [25].

This study showed that UTI was the most common type of infection, followed by sepsis. The empirical use of antibiotics for UTI and sepsis was appropriate in 56% and 68% of patients, respectively. The high frequency of UTI was expected, as it was reported in a recent review article as the most common infection in Saudi Arabia [26]. As this study was conducted among inpatients, only the higher frequency of sepsis is explainable, as sepsis represents a significant indication of hospital admission. As reported previously, sepsis is a frequent cause of hospital admission, especially in ICU admissions [27,28].

Although *E. coli* was the microorganism causing most infections, it was appropriately treated in 54% of cases. The high frequency of UTI explains the high frequency of *E. coli*. As mentioned previously, *E. coli* is a major cause of UTI [26,29]. *S. aureus* was isolated in 14.8% of cases and appropriately treated in 66% of cases. Meanwhile, MRSAs were isolated in 5.8% of the cases and appropriately treated in 59%. These findings are aligned with those of the 10-year surveillance study of MRSA infections in Saudi Arabia, which stated that “*S. aureus* continues to cause multiple site infections with a relatively stable methicillin-resistance rate, but the isolation of MRSA from the community is increasing” [30].

The results of this study illustrated that the three most common comorbidities recorded in patients were diabetes, chronic renal disease, and hypertension. The most appropriate empirical use of antibiotic therapy was observed with chronic renal failure, asthma, and diabetes, and the least appropriate use was with hypertension. Diabetes mellitus poses a well-recognized risk of infection, as do chronic renal diseases. Hypertension, as a risk of atherosclerosis, plays a role in attenuating the blood supply to body parts, which in turn lowers the immune status and increases the risk of infection. Therefore, controlling chronic debilitating diseases will significantly reduce the possibility of infections. Moreover, these findings raise the question as to whether comorbidities influence the local antimicrobial susceptibility pattern and open a window for future research. In this study, the highest percentage of microbial sensitivity was observed for linezolid, vancomycin, and tigecycline. Unfortunately, the frequent empirical use of ciprofloxacin and ceftriaxone affected their local susceptibility patterns, and they showed sensitivity in only 42% and 50% of cases, respectively. Similarly, a review article concerned with UTI in the KSA reported the existence of a significant resistance rate against ciprofloxacin [26]. Although the highest appropriate empirical use and a higher antimicrobial susceptibility in this study were found for vancomycin, there is international concern about the inappropriate empirical use of vancomycin, which represents 24.9% of the total amount of vancomycin prescribed [31]. 

This study found that the appropriateness of the empirical use of antibiotics was significantly associated with the type of organism and antimicrobial susceptibility, rather than the type or site of infection. These findings suggest that concentrating on the expected type of organism and the local susceptibility pattern are more important in building local ASPs rather than depending solely on the site of infection. As reported internationally, the implementation of a local antimicrobial guide improves the appropriateness of antimicrobial use [32]. 

Although a cross-sectional study is a convenient method, it lacks proof of causality because it is performed in one period, which might have had an effect on this study. However, this study found valuable results that could assist in improving local and national ASPs.

## 4. Materials and Methods

Study design: A cross-sectional hospital-based study was conducted at a tertiary hospital (KFSH) in Qassim region of the Kingdom of Saudi Arabia (KSA) from January 2021 to January 2022. The appropriateness of the empirical use of antibiotics was determined based on their alignment with culture and sensitivity results. The empirical use of antibiotics using sensitivity results were categorized as appropriate, while the empirical use of antibiotics that showed resistance or intermediate resistance were considered inappropriate. The antibiotics were grouped according to the WHO AWaRe [10]. 

Study participants: The adequate sample size, as calculated by the G*power program (version 3.1.9), was 473 patients, and this study included 500 inpatients diagnosed with infections who received antibiotics empirically and had culture and sensitivity results. Patients with no bacterial growth in the culture and sensitivity testing and those with polymicrobial growth were excluded from this study.

Study setting: Data were collected from laboratory and inpatients’ records from KFSH using a data collection sheet that was divided into patient demographic, medical, and medication sections, including the empirical prescription of antibiotics, and the last section included the results of antimicrobial susceptibility patterns. 

Statistics: The observations from the records of the patients’ treatment histories were entered into an Excel spreadsheet (Windows 10). The variables were represented as numbers and percentages. The statistical analysis of the data was carried out using GraphPad InStat software version 9.2. The non-parametric chi-square test was utilized for the logistic regression analysis. The data from the analysis of the study were compared and indicated as significant if the *p*-value was less than 0.05.

Ethical consideration: This study was approved by the Qassim Region Research Ethics Committee, registered with the National Committee of Bio and Med. Ethics (NCBE), numbered 1441-1349836, and dated 26 February 2020.

## 5. Conclusions

This cross-sectional study investigated the appropriateness of empirical use of antibiotics for five hundred inpatients at KFSH. There were slightly more men than women. The majority were over 61 years of age. Empirical uses of antibiotics were appropriately prescribed in fifty-eight percent of the patients. Ciprofloxacin and ceftriaxone were the most frequently prescribed antibiotics, while vancomycin, piperacillin–tazobactam, and tigecycline were the most appropriately prescribed antibiotics. *E. coli* was the microorganism most commonly isolated and was appropriately prescribed in 59% of the patients. Microorganisms were highly sensitive to linezolid, vancomycin, and tigecycline. A more appropriate empirical use of antibiotics was noted for the type of organism and sensitivity testing, so concentrating on the expected type of organism and the local susceptibility pattern is more important in building local ASPs.

## Figures and Tables

**Table 1:** Demographic characteristics of patients undergoing empirical use of antibiotics.

Age	Sex		Total N (%)
	Male N (%)	Female N (%)	
Less than 18 years	5 (1)	5 (1)	10 (2)
18–40 years	66 (13.4)	51 (10.2)	117 (23.4)
41–60 years	92 (18.4)	66 (13.2)	158 (31.6)
More than 60 years	119 (23.8)	96 (19.2)	215 (43)
Total N (%)	282 (56.4)	218 (43.6)	500 (100)

**Table 2:** The frequency of the empirical use of antibiotics with the percentage of their appropriate prescription based on antimicrobial susceptibility results.

Antibiotics	WHO AWaRe Groups	Total N (%)	Appropriate N (%)	Inappropriate N (%)
Ciprofloxacin	Watch	56 (11.2)	28 (50.0)	28 (50.0)
Levofloxacin	Watch	18 (3.6)	8 (44.4)	10 (55.6)
Amoxicillin + clavulanic acid	Access	49 (9.8)	32 (65.3)	17 (34.7)
Ceftriaxone	Watch	52 (10.4)	32 (61.5)	20 (38.5)
Cefotaxime	Watch	11 (2.2)	6 (54.5)	5 (45.5)
Vancomycin	Watch	35 (7.0)	33 (94.3)	2 (5.7)
Oxacillin	Access	19 (3.8)	10 (52.6)	9 (47.4)
Piperacillin + tazobactam	Watch	33 (6.6)	24 (72.7)	9 (27.3)
Linezolid	Reserve	43 (8.6)	27 (62.8)	16 (37.2)
Ceftazidime	Reserve	49 (9.8)	24 (49.0)	25 (51.0)
Cefuroxime	Watch	6 (1.2)	3 (50.0)	3 (50.0)
Meropenem	Watch	33 (6.6)	18 (54.5)	15 (45.5)
Imipenem	Watch	22 (4.4)	3 (13.6)	19 (86.4)
Cefepime	Watch	21 (4.2)	11 (52.4)	10 (47.6)
Tigecycline	Reserve	11 (2.2)	8 (72.7)	3 (27.3)
Amikacin	Access	9 (1.8)	6 (66.7)	3 (33.3)
Cefazolin	Access	16 (3.2)	6 (37.5)	10 (62.5)
Metronidazole	Access	17 (3.4)	12 (70.6)	5 (29.4)
Total	-	500	291 (58)	209 (42)

**Table 3:** The appropriateness of the empirical use of antibiotics according to type of infection.

Type of Infection	Total N (%)	Alignment of Empirical Use of Antibiotic with Antimicrobial Susceptibility Results	
		Appropriate N (%)	Inappropriate N (%)
UTI	123 (46.6)	69 (56)	54 (44)
Sepsis	100 (20)	68 (68)	32 (32)
Abscess	76 (15.2)	35 (46)	41 (54)
LRTI	69 (13.8)	50 (72)	19 (28)
Skin infection	53 (10.6)	26 (49)	27 (51)
Diabetic ulcer	25 (5)	12 (48)	13 (52)
URTI	23 (4.6)	15 (65)	8 (35)
CNS infection	18 (3.6)	8 (44)	10 (56)
Abdominal infection	8 (1.6)	6 (75)	2 (25)
Burn	5 (1)	2 (40)	3 (60)
Total	500	291 (58)	209 (42)

Urinary tract infection (UTI), lower respiratory tract infection (LRTI), upper respiratory tract infection (URTI), central nervous system (CNS).

**Table 4:** The appropriateness of the empirical use of antibiotics according to the type of microorganism.

Microorganism	Total N (%)	Alignment of Empirical Use of Antibiotic with Antimicrobial Susceptibility Results	
		Appropriate N (%)	Inappropriate N (%)
*Escherichia coli*	167 (33.4)	90 (54)	77 (46)
*Staphylococcus aureus*	74 (14.8)	46 (62)	28 (38)
*Acinetobacter baumannii*	66 (13.2)	46 (70)	20 (30)
*Enterococcus avium*	42 (8.4)	21 (50)	21 (50)
*Enterobacter cloacae*	28 (5.6)	16 (57)	12 (43)
MRSA	27 (5.4)	16 (59)	11 (41)
*Citrobacter koseri*	20 (4)	12 (60)	8 (40)
*Acinetobacter lwoffii*	14 (2.8)	9 (64)	5 (36)
*Pseudomonas aeruginosa*	13 (2.6)	7 (54)	6 (46)
*Proteus mirabilis*	12 (2.4)	7 (58)	5 (42)
*Streptococcus agalactiae*	12 (2.4)	6 (50)	6 (50)
*Klebsiella pneumonia*	10 (2)	5 (50)	5 (50)
*Enterobacter aerogenes*	8 (1.6)	6 (75)	2 (25)
*Diphtheroids* *	7 (1.4)	4 (57)	3 (43)
Total	500	291 (58)	209 (42)

Methicillin-resistant Staphylococcus aureus (MRSA); * Diphtheroids include Corynebacterium pseudotuberculosis, C. ulcerans, and C. haemolyticum.

**Table 5:** The appropriateness of the empirical use of antibiotic according to comorbidity.

Comorbidities	Total (N = 261) N (%)	Empirical Use of Antibiotics	
		Appropriate N (%)	Inappropriate N (%)
Hypertension	52 (19.9)	32 (61.5)	20 (38.5)
Diabetes	93 (35.6)	81 (87)	12 (13)
Asthma	16 (6.1)	14 (87.5)	2 (12.5)
Chronic renal disease	61 (23.4)	54 (88.5)	7 (11.5)
Congestive heart failure	7 (2.7)	5 (71.4)	2 (28.6)
Ischemic heart disease	14 (5.4)	10 (71.4)	4 (28.6)
Hypothyroidism	11 (4.2)	9 (82)	2 (18)
Epilepsy	7 (2.7)	6 (85.7)	1 (14.3)

**Table 6:** The antimicrobial susceptibility patterns of different pathogens.

Tested Antibiotic	WHO AWaRe Group	Sensitivity Testing		
		Total	Sensitive N (%)	Resistance N (%)
Imipenem	Watch	367	212 (58)	155 (42)
Cefepime	Watch	375	159 (42)	216 (58)
Oxacillin	Access	29	14 (48)	15 (52)
Cefuroxime	Watch	227	114 (50)	113 (50)
Tigecycline	Reserve	144	133 (92)	11 (8)
Ceftriaxone	Watch	122	61 (50)	61 (50)
Ciprofloxacin	Watch	444	187 (42)	257 (58)
Linezolid	Reserve	88	86 (98)	2 (2)
Levofloxacin	Watch	317	155 (49)	162 (51)
Vancomycin	Watch	100	96 (96)	4 (4)
Meropenem	Watch	385	239 (60)	146 (40)
Ceftazidime	Reserve	362	148 (41)	214 (59)
Cefotaxime	Watch	238	119 (50)	119 (50)

## Data Availability

The data presented in this study are available on request from the corresponding author due to local hospital regulations.
